# Collagenous Colitis in a Young Adult: A Case Report

**DOI:** 10.7759/cureus.101011

**Published:** 2026-01-07

**Authors:** João Lagarteira, Rita Pera, Sara Remelhe Sá, Ana Figueiredo, Andrés Carrascal

**Affiliations:** 1 Internal Medicine Department, Unidade Local de Saúde do Nordeste, Bragança, PRT

**Keywords:** collagenous colitis, colonic biopsies, microscopic colitis, subepithelial collagen band, watery diarrhea

## Abstract

Collagenous colitis is a subtype of microscopic colitis characterized by chronic watery diarrhea, normal-appearing colonic mucosa on endoscopy, and a thickened subepithelial collagen band on histological examination. Although it is often observed in middle-aged women, increasing recognition of this condition has expanded its epidemiological spectrum. We describe the case of a 21-year-old woman presenting with persistent watery diarrhea and weight loss. Extensive evaluation, including endoscopy with biopsies, confirmed collagenous colitis. This case highlights diagnostic and therapeutic considerations in young patients with chronic diarrhea. Although colonoscopy findings may be normal, further mucosal biopsies are essential for diagnosis. Budesonide remains the first-line treatment and achieves high remission rates. Collagenous colitis should be considered in the differential diagnosis of chronic watery diarrhea, even in young adults. Furthermore, early biopsy and appropriate therapy are crucial for symptom control and improved quality of life.

## Introduction

Collagenous colitis is a form of microscopic colitis, a group of inflammatory conditions of the colon that cause chronic, non-bloody watery diarrhea despite a normal or near-normal appearance on colonoscopy. The term microscopic reflects the fact that the diagnosis cannot usually be made by visual inspection alone and instead requires examination of tissue samples under a microscope. In collagenous colitis, this microscopic evaluation reveals a distinctive thickened layer of collagen, a structural protein, beneath the surface lining of the colon, along with inflammation in the surrounding connective tissue known as the lamina propria [1]. These changes interfere with normal fluid absorption in the colon, leading to persistent diarrhea that can significantly impair quality of life.

Patients with collagenous colitis often experience prolonged symptoms before diagnosis because routine endoscopic examinations may appear normal, leading to delayed or missed recognition of the disease. While collagenous colitis most commonly affects middle-aged and older women, it is increasingly recognized across a wider age range, including younger adults [2]. The condition has a multifactorial etiology involving immune system dysregulation, genetic susceptibility, and environmental influences. Several commonly prescribed medications, such as nonsteroidal anti-inflammatory drugs (NSAIDs), proton pump inhibitors (PPIs), and selective serotonin reuptake inhibitors (SSRIs), have been implicated as potential triggers or exacerbating factors [3]. In addition, collagenous colitis frequently coexists with other autoimmune disorders, including celiac disease and thyroid dysfunction, further complicating diagnosis and management [4].

Histologic evaluation of colonic biopsies remains the diagnostic gold standard, with the defining feature being a subepithelial collagen band measuring greater than 10 μm in thickness, accompanied by inflammatory cell infiltration [1,5]. Once diagnosed, treatment with budesonide has been shown to be highly effective in inducing both clinical and histologic remission [6,7].

Although collagenous colitis in younger patients has been previously described, particularly in the presence of known risk factors such as smoking or medication exposure, diagnostic delay remains common in this population due to lower clinical suspicion. This case underscores the ongoing importance of maintaining awareness of microscopic colitis across age groups and reinforces the need for routine colonic biopsies in patients with chronic watery diarrhea, even when colonoscopic findings are unremarkable.

## Case presentation

A 21-year-old woman presented with several months of watery, non-bloody diarrhea, mild diffuse abdominal discomfort, and unintentional weight loss of approximately 6 kg. She reported no fever, vomiting, recent antibiotic use, travel, or infectious exposures. Her medical history included having a single left kidney (probably a multicystic right kidney that was reabsorbed) and obesity. She was not under any regular medication.

On examination, the patient appeared well, with stable vital signs: blood pressure 115/65 mmHg, heart rate 97 bpm, temperature 36.7°C, and oxygen saturation 99% on room air. The abdominal exam was unremarkable, with no tenderness or organomegaly reported.

Laboratory results are provided in Table 1. Complete blood count was within normal limits. Mildly elevated inflammatory markers (C-reactive protein, 4.57 mg/L; erythrocyte sedimentation rate, 71 mm). Liver enzymes were slightly elevated (aspartate aminotransferase, 41 U/L; alanine aminotransferase, 46 U/L). Electrolytes, renal function, thyroid function, and coagulation studies were normal. Autoimmune screening was unremarkable. Infectious stool studies, including Clostridioides difficile toxin assays, were negative.

**Table 1:** Laboratory findings after comprehensive evaluation ALP: alkaline phosphatase; ALT: alanine transaminase; ANA: antinuclear antibodies; ANCA: autineutrophil cytoplasmic antibody; AST: aspartate aminotransferase; CK: creatine kinase; CRP: c-reactive protein; ESR: erythrocyte sedimentation rate; GGT: gamma-glutamyl transferase; HBV: hepatitis B virus; HCV: hepatitis C virus; HIV: human immunodeficiency virus; INR: international normalized ratio; LDH: lactate dehydrogenase; MPO: myeloperoxidase; PR3: proteinase 3; TSH: thyroid-stimulating hormone; T4: thyroxine

Parameters	Patient Values	Reference Range
Hemoglobin (g/dL)	13.9	12.3-15.3
Total leucocyte count (×10^9^/L)	5.03	4.4-11.3
Differential leucocyte count (%)	-	-
Neutrophils	64.1	50-70
Lymphocytes	27.0	25-40
Monocytes	7.5	2-8
Eosinophils	0.8	1-4
ESR (mm)	71	4.0-11.0
Platelet count (x10^9^/L)	271	150-450
INR	1.02	-
Sodium (mEq/L)	139	137-145
Potassium (mEq/L)	4.1	3.5-5.1
Chloride (mEq/L)	103	98-107
Glucose (mg/dL)	103	74-106
Urea (mg/dL)	18	17-43
Creatinine (mg/dL)	1.0	0.66-1.09
ALT (U/L)	46	<45
AST (U/L)	41	<35
Total bilirubin (mg/dL)	0.43	0.3-1.2
Direct bilirubin (mg/dL)	0.10	<0.2
ALP (U/L)	50	30-120
GGT (U/L)	36	<38
LDH (U/L)	207	<248
CK (U/L)	62	<145
CRP (mg/dL)	4.57	<0.1
TSH (uUI/mL)	2.18	0.35-4.94
Free T4 (ng/dL)	0.96	0.7-1.48
HIV	Negative	-
HBV	Negative	-
HCV	Negative	-
ANA (UI/mL)	0.1	<0.7=Negative
ANCA MPO (UI/mL)	<0.01	<3.5=Negative
ANCA PR3 (UI/mL)	<0.01	<2.0=Negative
Tissue transglutaminase (tTG) IgA, human recombinant (U/mL)	0.3	<7=Negative
Deamidated gliadin peptide IgG (U/mL)	<0.01	<7=Negative

**Figure 1:**
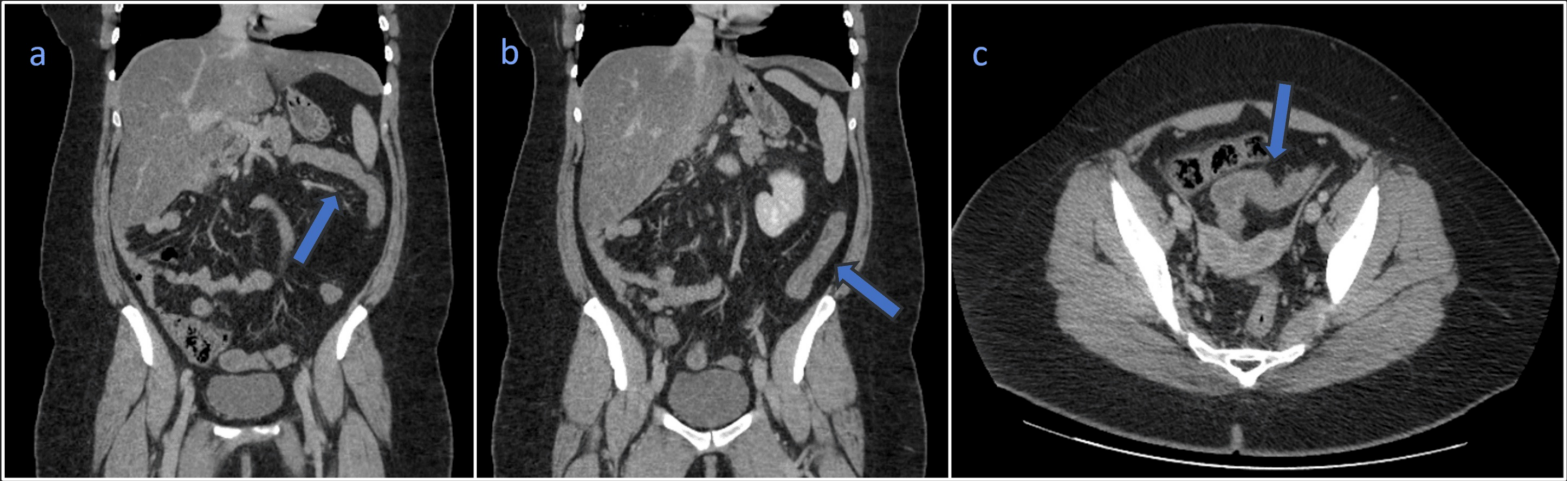
Abdominopelvic computed tomography scan (a) Coronal view showing signs of colitis in the splenic flexure; (b) Coronal view showing signs of colitis in the descending colon; (c) Axial view showing signs of colitis in the sigmoid colon.

Abdominopelvic computed tomography demonstrated features suggestive of colitis, involving the splenic, descending, and sigmoid flexures, manifested by mild parietal thickening, with submucosal edema, mucosal hyperuptake, mild pericolic edema, with no evidence of obstruction or complications (Figure 1).

**Figure 2:**
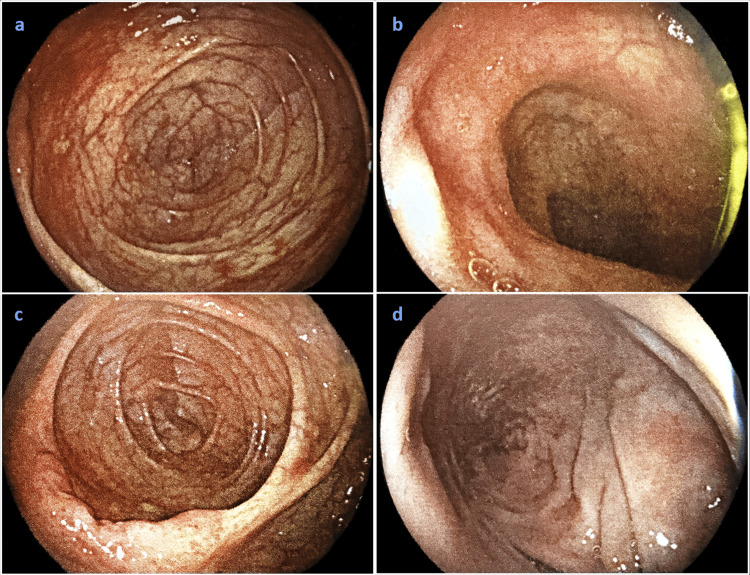
Colonoscopy Images from a colonoscopy in sequence (a-d), revealing normal mucosa, aside from mild edema and a decreased vascular pattern.

Colonoscopy revealed normal mucosa, aside from mild edema and a decreased vascular pattern (Figure 2). Systematic biopsies were taken along the colon.

Histological examination revealed a prominent subepithelial collagen band greater than 10 μm, along with mononuclear inflammatory infiltration in the lamina propria, findings indicative of collagenous colitis. Gastric biopsies revealed mild chronic gastritis without detecting *Helicobacter pylori*.

Prior to the biopsy results, the patient was empirically treated with intravenous ceftriaxone and metronidazole, without clinical improvement. Upon confirmation of collagenous colitis, treatment with oral budesonide was initiated (9 mg/day). The patient experienced rapid resolution of diarrhea within 1 week. Budesonide was tapered over 12 weeks, and the patient remained asymptomatic at the 6-month follow-up.

## Discussion

Collagenous colitis is classically described as a disease of middle-aged and older women; however, an increasing number of reports have documented its occurrence in younger adults [2]. While this demographic shift is now well recognized, younger patients are still at risk of delayed diagnosis due to lower clinical suspicion and the frequent attribution of chronic watery diarrhea to functional bowel disorders. In our patient, the combination of persistent symptoms and a normal-appearing colonic mucosa exemplifies this diagnostic challenge and reinforces the importance of maintaining microscopic colitis in the differential diagnosis regardless of age.

Chronic watery diarrhea has a broad differential diagnosis that includes irritable bowel syndrome, celiac disease, bile acid diarrhea, inflammatory bowel disease, medication-induced enteropathy, and chronic infections. Unlike many of these conditions, microscopic colitis cannot be reliably excluded based on endoscopic findings alone, as the colonic mucosa often appears macroscopically normal [5,8]. Current guidelines therefore recommend systematic biopsies from multiple colonic segments to detect the hallmark histologic features, particularly subepithelial collagen deposition in collagenous colitis [9]. In this context, our case illustrates the diagnostic value of routine biopsies and highlights how reliance on endoscopic appearance alone may contribute to underdiagnosis.

Medication exposure, most notably NSAIDs, PPIs, and SSRIs, is a well-established risk factor for microscopic colitis and is frequently implicated in younger patients [3]. Autoimmune comorbidities, including celiac disease, are also more prevalent in this age group and may contribute to disease development [4]. In contrast to what is commonly reported in the literature, our patient had no identifiable medication triggers or autoimmune conditions, suggesting an idiopathic presentation. This distinction is clinically relevant, as it underscores that the absence of recognized risk factors does not exclude the diagnosis and should not deter clinicians from pursuing histologic evaluation when symptoms persist.

Budesonide remains the first-line therapy for both collagenous and lymphocytic colitis, with clinical remission rates exceeding 80% in randomized controlled trials [6,7,10]. Our patient’s rapid clinical response is consistent with these findings and further supports the effectiveness of budesonide as induction therapy. Nevertheless, relapse after treatment discontinuation is common, occurring in a substantial proportion of patients, and may necessitate prolonged or maintenance therapy [10].

From an analytical perspective, this case contributes to the existing literature by illustrating that collagenous colitis can present idiopathically in younger adults, respond promptly to standard therapy, and achieve sustained remission with appropriate tapering. More importantly, it highlights a persistent gap between guideline recommendations and real-world diagnostic practice, particularly the underutilization of routine biopsies in patients with chronic diarrhea and normal colonoscopy findings.

This case emphasizes the need for heightened clinical awareness of collagenous colitis across age groups and reinforces the principle that normal endoscopic findings do not obviate the need for histologic assessment. Early recognition and appropriate treatment can lead to rapid symptom resolution, prevent unnecessary diagnostic delays, and significantly improve patient quality of life.

## Conclusions

This case demonstrates the importance of including collagenous colitis in the differential diagnosis of chronic watery diarrhea, even among young adults, a population in which the condition may be less frequently diagnosed. Normal colonoscopic findings do not exclude microscopic colitis, and routine biopsies should be obtained when symptoms persist and the initial investigation is inconclusive. Our findings further support existing evidence that histopathologic confirmation, specifically the presence of a thickened subepithelial collagen band and inflammatory infiltrates, remains the diagnostic gold standard. Consistent with current literature, budesonide proved to be an effective first-line treatment, leading to clinical remission and symptom control. Clinicians should maintain awareness of recognized risk factors in younger patients, including smoking, certain medications such as NSAIDs, PPIs, and SSRIs, or persistent gastrointestinal symptoms despite prior interventions, to facilitate earlier diagnosis, prevent unnecessary investigations or ineffective treatments, and improve quality of life. This case illustrates a textbook presentation in a young woman and underscores the value of early recognition and histologic confirmation in optimizing patient outcomes.
